# Validity of Three IRT Models for Measuring and Controlling Extreme and Midpoint Response Styles

**DOI:** 10.3389/fpsyg.2020.00271

**Published:** 2020-02-21

**Authors:** Yingbin Zhang, Yehui Wang

**Affiliations:** ^1^Collaborative Innovation Center of Assessment for Basic Education Quality, Beijing Normal University, Beijing, China; ^2^The Department of Curriculum and Instruction, College of Education, University of Illinois at Urbana-Champaign, Champaign, IL, United States

**Keywords:** mixed partial credit model, multidimensional nominal response model, item response tree model, extreme response style, midpoint response style

## Abstract

Response styles, the general tendency to use certain categories of rating scales over others, are a threat to the reliability and validity of self-report measures. The mixed partial credit model, the multidimensional nominal response model, and the item response tree model are three widely used models for measuring extreme and midpoint response styles and correcting their effects. This research aimed to examine and compare their validity by fitting them to empirical data and correlating the content-related factors and the response style-related factors in these models to extraneous criteria. The results showed that the content factors yielded by these models were moderately related to the content criterion and not related to the response style criteria. The response style factors were moderately related to the response style criteria and weakly related to the content criterion. Simultaneous analysis of more than one scale could improve their validity for measuring response styles. These findings indicate that the three models could control and measure extreme and midpoint response styles, though the validity of the mPCM for measuring response styles was not good in some cases. Overall, the multidimensional nominal response model performed slightly better than the other two models.

## Introduction

Response styles refer to the systematic preference or avoidance of certain response categories in assigning ratings to personality and attitudinal items (Paulhus, 1991). They are one of the major sources of common method bias (Podsakoff et al., 2012). There are four main response styles: the acquiescence response style (ARS), which is the preference to select categories stating agreement; the disacquiescence response style (DARS), which is the preference to select categories stating disagreement; the extreme response style (ERS), which is the tendency to select extreme categories; and the midpoint response style (MRS), which is the tendency to select the middle category or neutral category.

Past research has revealed that response styles may be conceptualized as trait-like constructs that are stable across time (Weijters et al., 2010b), consistent across scales (Wetzel et al., 2013), and related to respondent characteristics, such as personality traits (Naemi et al., 2009). They can cause biased scale scores (Moors, 2012; Mottus et al., 2012) and correlations (e.g., Baumgartner and Steenkamp, 2001), and underestimation of measurement invariance (e.g., Liu et al., 2017).

Some approaches have been proposed to measure and control response styles. For example, one traditional method for controlling ARS uses a balanced scale with positively and negatively keyed items (Paulhus, 1991). Together with confirmatory factor analysis, this method can also measure ARS (Billiet and Davidov, 2008). Moors (2012), Moors et al. (2014) extended the confirmatory latent class analysis to measure and control ARS and ERS. The method of representative indicators for response styles have been used to measure all the main response styles (Greenleaf, 1992; Weijters et al., 2010b), but it usually requires many items (Jin and Wang, 2014).

More approaches are within the item response theory (IRT) framework. Among them, three IRT models and their extensions are widely applied in practice: the mixed partial credit models (mPCM; Rost, 1991), the multidimensional nominal response model (MNRM; Bolt and Johnson, 2009), and the item response tree model (IR tree model; Böckenholt, 2012; De Boeck and Partchev, 2012). These models separate responses to items into response style variance and content variance and produce response style-related latent factors as measures of response styles and content-related latent factors as measures of content free of response style effects. However, these approaches have different assumptions about the nature of response styles and the response process. The current research examined and compared their validity for measuring ERS and MRS and correcting content-related factor scores for ERS and MRS effects.

### Mixed Partial Credit Models for Response Styles

Mixed partial credit models (Rost, 1991) are extensions of the partial credit model (PCM; Masters, 1982). In the mPCM, item and person parameters are estimated separately for each latent class, and thus we can investigate the qualitative differences between the latent classes. Rost et al. (1997) discussed the identification of latent subpopulations with different response styles. As they pointed out, the avoidance of extreme categories in some subpopulations is indicated by a large negative first threshold and a large positive last threshold.

Let there be *k* = 1, …, *N* possible ordered categories and *c* = 1, …, *C* latent classes. The probability of a response in the category *k* of item *i* is modeled as

$$\text{P}\left(Y_{i}=k|\theta ,\delta \right)=\sum_{c=1}^{C}\pi_{c}\frac{\exp\,\sum_{r=1}^{k}\left(\theta_{c}-\delta_{i\,r\,c}\right)}{\sum_{h=1}^{N}e\,x\,p\,\sum_{r=1}^{h}\left(\theta_{c}-\delta_{i\,r\,c}\right)}\;\left(1\right)$$

**θ**_*c*_ and **δ**_*irc*_ are class-specific person and threshold parameters. **π**_*c*_ denotes the probability of class *c*, and $\sum_{c=1}^{C}\pi_{c}=1$. The mPCM cannot account for individual differences in response styles within a subpopulation. Thus, when we use the mPCM to measure response styles, it assumes that response styles vary across subpopulations but not within a subpopulation.

### Multidimensional Nominal Response Models for Response Styles

Bolt and Johnson (2009) proposed a multidimensional extension of Bock (1972) nominal response model to model the extreme response style (ERS) and correct its effect. Their approach was exploratory because the category slope parameters were estimated rather than specified. Johnson and Bolt (2010) utilized the multidimensional nominal response model (MNRM) with specified category slope parameters to measure ERS. Then, the work promoted by Bolt and Johnson was extended by Wetzel and Carstensen (2017), making the MNRM also appropriate for measuring other kinds of response styles, such as midpoint response style (MRS). Let there be *k* = 1, …, *N* possible ordered response categories, and *d* = 1, …, *d* latent dimensions. Under the MNRM, the probability of a response in category *k* of item *i* is modeled as

$$\text{P}\left(Y_{i}=k|\text{a},\theta ,\text{b}\right)=\frac{e\,x\,p\,\left(a_{i\,k\,1}\,\theta_{1}+a_{i\,k\,2}\,\theta_{2}+\cdots +a_{i\,k\,D}\,\theta_{D}+b_{i\,k}\right)}{\sum_{h=1}^{N}e\,x\,p\,\left(a_{i\,h\,1}\,\theta_{1}+a_{i\,h\,2}\,\theta_{2}+\cdots +a_{i\,h\,D}\,\theta_{D}+b_{i\,h}\right)}\;\left(2\right)$$

*a* and *b* represent the category slope parameter and the category intercept parameter, respectively. By specifying category slope parameters rather than letting them be freely estimated, we can use the MNRM to separate content and response styles. Assuming that *K* = 5 and that we need to model the content factor, ERS and MRS, we can apply the constraints or scoring functions depicted in Table 1. Then, **θ**_*1*_ denotes the content factor, while **θ**_*2*_ and **θ**_*3*_ denote the ERS factor and the MRS factor, respectively. The MNRM is a compensatory multidimensional model, so it assumes that the response style factor can compensate for the content factor when using the MNRM to separate content and response styles.

**TABLE 1:** Scoring functions for the content factor, ERS and MRS.

Category	1	2	3	4	5
*a*_*k1*_	0	1	2	3	4
*a*_*k2*_	1	0	0	0	1
*a*_*k3*_	0	0	1	0	0

*Response category *k* (*k* = 1, 2, 3, 4, 5).*

### Item Response Tree Models for Response Styles

Böckenholt (2012) and De Boeck and Partchev (2012) presented the IR tree model to model multiple processes involved in responses to polytomous items. For example, responses to a 5-point item involves three processes (Table 2). Process I results in the choice of the middle point (3) with probability P_1_ or process II with probability 1 – P_1_. Process II results in agreement (4, 5) with probability P_2_ or disagreement (1, 2) with probability 1 – P_2_. Then, process III is activated and leads to a choice of extreme categories (1 or 5) with probability P_3_, or to a choice of non-extreme categories (2, 4) with probability 1 – P_3_. Thus, the content factor is involved in process II, while the MRS factor and the ERS factor are involved in process I and process III, respectively.

**TABLE 2:** The decomposition method of a 5-point item.

Response category	1	2	3	4	5
Pseudo item I/Process I	0	0	1	0	0
Pseudo item II/Process II	0	0	–	1	1
Pseudo item III/Process III	1	0	–	0	1

Based on these processes, researchers need to decompose a 5-point item into three binary pseudo items in order to apply the IR tree model to separating content and response styles. Pseudo items for the same process can be fitted by a dichotomous IRT model (either one parameter or two parameters).

The IR tree model is similar to Thissen-Roe and Thissen (2013) two-decision model. When the number of response categories is four, the two models are identical when modeling ERS. However, when there are more than four categories, the two-decision model decomposes an item into polytomous items and fits the polytomous item with a modified version of Samejima’s (1969) graded model (see Thissen-Roe and Thissen, 2013).

### Current Research

The rationales for these approaches differ greatly. In the mPCM, response styles vary across latent subpopulations but are consistent within a subpopulation, and thus, response styles are categorical variables. In contrast, response styles are continuous variables in the MNRM and IR tree models. However, the two models have different assumptions about how response styles influence responses to items jointly with content factors. The MNRM assumes that the response styles compensate for the content factor, while the IR tree model assumes that response styles and the content factor function independently in different response processes.

Although these approaches for separating content and response styles are different, there are few empirical studies examining and comparing their efficacy. Plieninger and Meiser (2014) investigated the validity of the IR tree model, but they did not compare the IR tree model with the mPCM and MNRM. Böckenholt and Meiser (2017) and Leventhal and Stone (2018) did compare these models, but the former focused on the rationales, implementation, and estimation of the IR tree model and the mPCM, while Leventhal and Stone (2018) examined the item mean square error and model fit of the IR tree model and the MNRM. There is no study investigating whether the mPCM and MNRM can effectively measure ERS and MRS and correct their effects in empirical data. Besides, it is unclear that among the three models, which one performs best. The current research aims to address this gap.

This article examines and compares the validity of these models through two empirical studies. These studies adopted Plieninger and Meiser (2014) research paradigm: utilizing extraneous criteria for content and response styles to examine and compare the validity of these approaches. Depending on the content, there may or may not be an expected correlation between response style factors and the content criterion, as well as between the content factor and the response style criteria. However, if one model is valid for measuring response styles, response style factors in the model should be more related to the response style criteria than the content criterion. If one model is valid for correcting the content factor for response style effects, the association between the content factor and the response style criteria should be smaller in this model than in a model that does not consider response styles (e.g., the PCM).

The response style criteria were obtained with the representative indicators for response styles method (RIRS; Greenleaf, 1992; Weijters et al., 2008; De Beuckelaer et al., 2010). This approach was also used by Plieninger and Meiser (2014) as the response style criteria. It computes response style scores from a set of highly heterogeneous items to avoid content variance and thus is a valid and stable measure of response styles (De Beuckelaer et al., 2010). The detail of the response style criteria and the content criteria were described in each study.

## Study 1

Plieninger and Meiser (2014) used the IR tree model to separate content, ERS, and MRS in response to learning self-confidence items (the target instrument) and validating the IR tree model with extraneous criteria. Study 1 analyzed the same data to examine the validity of the mPCM and MNRM and comparing the three models^1^.

The data were from the 11th round of the Constance Survey of Students (Georg and Ramm, 2016). Participants received, answered and returned a print questionnaire during the winter of 2009. Twenty participants were excluded, nineteen for not responding to over half the items on the target instrument and one for random responding. The final sample consisted of 7,570 students, with 56.5% being female and a mean age of 24.11 years (*SD* = 4.49). There were 20 missing values for sex and 7 for age.

### Methods

To be consistent with Plieninger and Meiser (2014) research, Study 1 utilized the same target instrument and extraneous criteria.

#### Target Instrument

The target instrument was composed of nine items reflecting learning self-confidence at the university. There were seven response categories numbered from 0 (not at all) to 6 (totally true) without verbal labels in between. Three items were negatively worded and reversely scored prior to analyses. Cronbach’s α was 0.67.

#### Extraneous Criteria

As the target instrument reflected the aspects of learning self-confidence, which was related to academic performance, Plieninger and Meiser (2014) took academic grades as the content criterion. Academic grades were measured via one item that required students to indicate the current average of their university grades on the typical German scale ranging from 1 (excellent) to 6 (fail). The mean was 2.31 (*SD* = 0.61), and the item was recoded to make higher scores indicative of better performance.

The questionnaire in the 11th round of the Constance Survey of Students comprised more than 500 items about students’ lives. It allowed us to select many heterogeneous items as the response style criteria. Sixty items with a 7-point response format were chosen by Plieninger and Meiser (2014). Only the extreme categories of these items were labeled. The heterogeneity of these items was satisfactory, with an average absolute correlation of 0.06. The proportion of extreme responses (e.g., selecting category 1 or 7 when response categories ranged from 1 to 7) on the sixty items was used as the ERS criterion, and the proportion of midpoint responses (e.g., selecting category 4 when response categories ranged from 1 to 7) was used as the MRS criterion. The means of the ERS criterion and the MRS criterion were 0.29 (*SD* = 0.13) and 0.16 (*SD* = 0.07), respectively. Plieninger and Meiser (2014) had confirmed the validity of the response style criteria.

#### Implementation of the Three IRT Models

All models were estimated with maximum likelihood estimation with robust standard errors (MLR) in Mplus 7.11 (Muthén and Muthén, 2015). Twelve quadrature points were specified for numerical Gauss–Hermite integration. The link function was logit.

##### The mPCM

The two-class mPCM was the most frequently used in distinguishing subpopulations with different response styles (e.g., Wetzel et al., 2013; Böckenholt and Meiser, 2017). However, the number of response categories of learning self-confidence items was 7, and it was likely that there were subpopulations with MRS. The two-class mPCM might be not appropriate in the current case. Therefore, the mPCM with two to seven latent classes was compared to determine the number of latent classes. The consistent Akaike’s Information Criterion (CAIC; Bozdogan, 1987), which was suitable for comparing mixed IRT models with different classes (Cho, 2013), was used. Table 3 displays the goodness-of-fit statistics of the mPCM with different latent classes. The CAIC of the six-class mPCM was lowest, and thus, this mPCM was used for further analysis.

**TABLE 3:** The goodness-of-fit statistics of the mPCM with two to seven classes.

No. of latent classes	No. of Par.	LL	CAIC
Two	101	−116556.07	234115.33
Three	147	−115803.46	233067.00
Four	193	−115407.12	232731.21
Five	239	−115102.84	232579.54
Six	285	−114853.62	232537.99
Seven	331	−114712.30	232712.26

*LL, Log-Likelihood. CAIC, Consistent Akaike’s Information Criterion.*

The threshold parameters in equation 1 were specific in each latent class, but the mean of the threshold parameter for each item (i.e., the item difficulty parameter) was set equal across latent classes to ensure measurement invariance across latent classes (Millsap, 2012). The means and variances of mathematics self-efficacy were also set equal between latent classes to avoid confounding threshold heterogeneity with changes in mathematics self-efficacy (Böckenholt and Meiser, 2017). The person parameter in this mPCM represented the content factor, while the latent class factor was regarded as the response style factor.

##### The MNRM

Table 4 displays the scoring functions applied in the MNRM. With this scoring function, θ_*1*_ in equation 3 represents the learning self-confidence factor, while θ_*2*_ and θ_*3*_ denote the ERS factor and the MRS factor, respectively.

**TABLE 4:** Scoring functions for learning self-confidence, ERS and MRS.

Category	0	1	2	3	4	5	6	Factor
*a*_*k1*_	0	1	2	3	4	5	6	Self-confidence
*a*_*k2*_	1	0	0	0	0	0	1	ERS
*a*_*k3*_	0	0	0	1	0	0	0	MRS

*Response category *k* (*k* = 0, 1, …, 6).*

##### The IR tree model

Table 5 depicts the decomposition method for the IR tree model in Study 2, which was also used by Plieninger and Meiser (2014). According to their study, the latent factor functioning at process I was highly correlated with the one functioning at process IV (*r* = 0.89), and thus, the two factors should be identical and the sets of pseudo items I and IV were forced to load on the same latent factor, which represented the MRS-related factor (Plieninger and Meiser; 2014). Pseudo item II and III measured learning self-confidence and ERS, respectively. Given that item discrimination was constant in the mPCM and the MNRM, the one-parameter dichotomous IRT model (1PL model) was used to fit the same kind of pseudo items (see Table 5). θ_*1*_, θ_*2*_ and θ_*3*_ denoted the MRS-related factor, the content factor, and the ERS-related factor, respectively.

**TABLE 5:** Decomposition of learning self-confidence items into pseudo items.

Response category	0	1	2	3	4	5	6	Model
Pseudo item I/Process I	0	0	0	1	0	0	0	${\text{P}}_{1}\left(Y_{i\,1}=1\right)=\frac{e\,x\,p\,\left(\theta_{1}-\beta_{i\,1}\right)}{1-e\,x\,p\,\left(\theta_{1}-\beta_{i\,1}\right)}$
Pseudo item II/Process II	0	0	0	–	1	1	1	${\text{P}}_{2}\left(Y_{i\,2}=1\right)=\frac{e\,x\,p\,\left(\theta_{2}-\beta_{i\,2}\right)}{1-e\,x\,p\,\left(\theta_{2}-\beta_{i\,2}\right)}$
Pseudo item III/Process III	1	0	0	–	0	0	1	${\text{P}}_{3}\left(Y_{i\,3}=1\right)=\frac{e\,x\,p\,\left(\theta_{3}-\beta_{i\,3}\right)}{1-e\,x\,p\,\left(\theta_{3}-\beta_{i\,3}\right)}$
Pseudo item IV/Process IV	–	0	1	–	1	0	–	${\text{P}}_{4}\left(Y_{i\,4}=1\right)=\frac{e\,x\,p\,\left(\theta_{1}-\beta_{i\,4}\right)}{1-e\,x\,p\,\left(\theta_{1}-\beta_{i\,4}\right)}$

*Item *i* (*i* = 1, 2, …, 9).*

#### Assessing the Validity of Models

The extraneous criteria were correlated with the content factor, the ERS factor, and the MRS factor to assess the validity of the three IRT models. For the mPCM, we recoded the latent class factor into five dummy variables, regressed extraneous criteria on them and showed the *R*-square.

### Results and Discussion

Table 6 displays the results about the validity of the three models. In all models, a moderate association existed between the content factor and its criterion, and the coefficients were almost equal. A clear dissociation between the content factor and the ERS criterion was observed. There was a weak association between the content factor and the MRS criterion in the mPCM and MNRM, and there was a clear dissociation between the two variables in the IR tree model. For comparison, the associations between the criteria and learning self-confidence were also investigated using the PCM. The correlation between learning self-confidence and its criterion in the PCM (0.56) were virtually identical to that in the mPCM (regardless of the number of latent classes), the MNRM and the IR tree model (0.54, 0.55, 0.53). Meanwhile, the correlation between the ERS criterion and learning self-confidence in the PCM (0.17) disappeared in the other IRT models (0.01, −0.03, 0.04), and the correlation between learning self-confidence and the MRS criterion in the PCM (−0.15) became weaker in the three models (−0.09, −0.10, −0.08). Overall, the results indicated that the three IRT models were valid for correcting the learning self-confidence factor for response style effects but did not change its relationship with its criterion.

**TABLE 6:** Relationships of criteria with the factors of the IRT models in Study 2.

*r* (*SE*)	Learning self-confidence			ERS factor		MRS factor		Latent classes of mPCM
				
	mPCM	MNRM	IR tree model	MNRM	IR tree model	MNRM	IR tree model	
Academic grades	0.54 (0.02)***	0.55 (0.01)***	0.53 (0.01)***	0.08(0.02)***	0.18(0.01)***	−0.19(0.03)***	−0.34(0.02)**	0.18(0.01)^a^***
ERS criterion	0.01 (0.02)	−0.03(0.01)	0.04 (0.02)	0.65 (0.01)***	0.64 (0.01)***	−0.23(0.04)***	−0.40(0.02)***	0.43 (0.01)^a^***
MRS criterion	−0.09(0.02)***	−0.10(0.02)***	−0.08(0.02)***	−0.20(0.02)***	−0.22(0.02)***	0.58 (0.05)***	0.39 (0.02)***	0.10 (0.01)^a^***

a, the *R*-square of regressing extraneous criteria on the latent class factor. The gray cell is the correlation between a factor and its criterion, or the *R*-square of regressing response style on the latent class factor. ***p < 0.001.

The ERS factors in the MNRM and the IR tree model were strongly related to the ERS criterion and weakly related to the content criterion. In addition, there was a negative moderate association between the MRS criterion and the ERS factor in the two IRT models. This association was in line with the actual relationship between ERS and MRS given that the ERS criterion was negatively related to the MRS criterion (−0.40). Thus, the MNRM and IR tree models were effective at extracting the ERS factor from the responses to the learning self-confidence items.

The MRS factor in the MNRM was positively and strongly related to the MRS criterion and negatively related to the ERS criterion. The weak association between the MRS factor and the content criterion was accepted because there was also a weak association between the MRS criterion and the content criterion (*r* = −0.11). This finding confirmed the validity of the MNRM for measuring MRS.

However, the IR tree model did not show such validity. The MRS factor in the IR tree model was only moderately related to its criterion, and the correlation coefficient (0.36) was even slightly lower than the absolute value of the correlation coefficient between the MRS factor and the ERS criterion (0.39). The reason might be that the definition of MRS as specified by the decomposition method in Table 5 was not in line with the MRS criterion; the MRS criterion was only the proportion of midpoint responses on the heterogeneous items (see section “Extraneous Criteria”), but in Table 5, both pseudo item I and item IV, which involved the responses to all of the three inner categories (2, 3, 4) rather than only the midpoint category (3), measured MRS. To test this speculation, a new decomposition method without pseudo item IV was implemented, and the corresponding IR tree model was estimated. The results confirmed the speculation: the correlation coefficient between the MRS factor and its criterion increased to 0.48, and the other relationships were virtually unaffected (−0.35 and −0.41 for the relationship between the MRS factor and the content criterion and the ERS criterion, respectively). This finding suggested that different methods for decomposing items were suitable for different definitions of MRS^2^. Nevertheless, the moderate association between the MRS factor and the content criterion indicated that the MRS factor contained some content variance, given that only a weak association was between the MRS criterion and the content criterion (*r* = −0.11). Thus, the validity of the IR tree model for measuring MRS was poorer than the MNRM.

For the mPCM, the association between the latent class factor and the ERS criterion (*R*-square = 0.43) was comparable with its counterpart in the MNRM and the IR tree model, but the association between the latent class factor and the MRS criterion (*R*-square = 0.10) was weaker than its counterpart in the MNRM and the IR tree model. As there is a moderate association between the ERS and MRS, the weak association between the latent class factor and the MRS criterion might indicate that the latent class factor only captured the variance of ERS and not suitable for measuring MRS. Moreover, the latent class factor was moderately related to the content criterion (*R*-square = 0.18).

Given that the mPCM implies greater measurement error in the response styles estimates than the IR tree model (Adams et al., 2019), and the mPCM did perform worse than the IR tree model in measuring MRS, the classification quality of the mPCM was checked to examine whether the classification quality might cause the poor efficacy of the mPCM. Two measures were used: the entropy and the reduction of classification error (see Supplementary Appendix; Vermunt, 2010). The closer they are to 1, the better the classification quality of the mPCM. The entropy and the reduction of classification error were 0.626 and 0.679, respectively, suggesting the low classification performance of the mPCM. The unsatisfactory classification accuracy might be the reason for the low performance of the mPCM in measuring MRS.

To summarize, the three models showed similar validity for correcting the effect of ERS and MRS. They could also measure these response styles, but the efficacy of the MNRM in measuring MRS was better than the IR tree model and the mPCM.

## Study 2

In Study 1, the associations between response style factors in the models and their criteria were not strong, and there were also weak or moderate associations between them and the content criteria. The reason might be that these models were naturally less effective at measuring response styles when dealing with one short scale each time (Bolt and Newton, 2011). Therefore, the main goal of Study 2 was to investigate whether the validity of these models for measuring response styles would increase when dealing with two scales.

Data was from the China-Shanghai 2012 PISA sample. The PISA 2012 Student Questionnaire had three forms because of the rotation design in the student questionnaire (OECD, 2014). It requires many heterogeneous items to obtain the criteria of ERS, but the number of common items between any two questionnaire forms was too small to extract adequate heterogeneous items from common item sets. Given that questionnaire form B had the most items, only students who responded to this questionnaire were included in the analysis. Five students were excluded due to not responding to over half items of the target instrument, the Mathematics Self-Efficacy Scale. The final sample consisted of 1,725 students from 156 schools, with 49.8% being female and a mean age of 15.80 (*SD* = 0.30).

In order to examine whether using the three models to analyze two scales simultaneously would increase their validity, Study 2 firstly applied them to analyzing only the Mathematics Self-Efficacy Scale, and then, applied them to analyzing both this scale and the Mathematics Anxiety scale.

### Methods

#### Target Instrument

The Mathematics Self-Efficacy Scale comprised eight items with a 4-point response format. Category labels for the items were “1 = strongly agree,” “2 = agree,” “3 = disagree,” and “4 = strongly disagree.” Items were recoded so that a higher score corresponded to a higher level of self-efficacy. The Cronbach’s α was 0.92.

#### Mathematics Anxiety Scale

This scale contained five items, and the response format and categories were the same as the mathematics self-efficacy scale. Items were recoded. The Cronbach’s α was 0.87.

#### Extraneous Criteria

PISA 2012 contained a mathematics achievement test that was analyzed using IRT. Instead of estimating a single person score, the analyses yielded five plausible values (PVs) of mathematics performance. As the mathematics self-efficacy and mathematics anxiety were associated with mathematics performance, respectively (e.g., Dowker et al., 2016), the five PVs were used as the content criteria. The “multiple imputation” option of Mplus was applied when the analysis included the PVs.

The proportion of extreme responses to thirty heterogeneous items was used as the ERS criterion. The items were chosen from the Likert items of questionnaire form B, excluding mathematics self-efficacy and anxiety items. The number of response categories of these items was four, and all categories were labeled. The average absolute item correlation among the items was 0.08, indicating high heterogeneity. The mean of the ERS criterion was 0.39 (*SD* = 0.17). The Cronbach’s α of recoded items (recoding 1/4 into 1 and 2/3 into 0) was 0.79.

#### Implementation of the Three IRT Models

The methods of estimation were the same as Study 1.

##### The mPCM

As the mathematics self-efficacy scale did not have a neutral category, only the effect of ERS existed in the data. Subpopulations with and without ERS were usually distinguished with the two-class mPCM (e.g., Wetzel et al., 2013; Böckenholt and Meiser, 2017). Therefore, the two-class mPCM was applied, and the latent class factor was seen as the ERS factor. The rest of the implementation was the same as Study 1.

##### The MNRM

To model mathematics self-efficacy and ERS with the MNRM, the scoring functions in Table 7 was applied. θ_*1*_ and θ_*2*_ denote the mathematics self-efficacy factor and the ERS factor, respectively.

**TABLE 7:** Scoring functions for mathematics self-efficacy and ERS.

Category	1	2	3	4	Factor
*a*_*k1*_	0	1	2	3	Self-efficacy
*a*_*k2*_	1	0	0	1	ERS

*Response category *k* (*k* = 1, 2, 3, 4).*

##### The IR tree model

Each item of the target instrument was recoded into two binary pseudo items (see Table 8). Pseudo items I and II measured mathematics self-efficacy and ERS, respectively.

**TABLE 8:** Decomposition of the mathematics self-efficacy items into pseudo items.

Category	1	2	3	4	Model
Pseudo item I/Process I	0	0	1	1	$P_{1}\left(Y_{i\,1}=1\right)=\frac{e\,x\,p\,\left(\theta_{1}-\beta_{i\,1}\right)}{1-e\,x\,p\,\left(\theta_{1}-\beta_{i\,1}\right)}$
Pseudo item II/Process II	1	0	0	1	$P_{2}\left(Y_{i\,2}=1\right)=\frac{e\,x\,p\,\left(\theta_{2}-\beta_{i\,2}\right)}{1-\exp\left(\theta_{2}-\beta_{i\,2}\right)}$

*Item *i* (*i* = 1, 2, …, 8).*

### Results and Discussion

Sampling weights (final student weights in the data set) were used to adjust for different sampling probabilities of students and to estimate results that could represent the population (cf. Rutkowski et al., 2010). Table 9 shows the relationships between factors in the models and the extraneous criteria.

**TABLE 9:** Relationships of criteria with the factors of IRT models in Study 2.

*r* (*SE*)	Mathematics self-efficacy			ERS factor		
		
	mPCM	MNRM	IR tree model	mPCM	MNRM	IR tree model
**Analyzing one scale**						
Mathematics achievement	0.58 (0.02)***	0.64 (0.03)***	0.67 (0.02)***	0.32(0.02)^a^***	0.28(0.03)***	0.51(0.02)***
ERS criterion	0.15(0.03)***	0.01 (0.04)	0.11(0.03)**	0.27 (0.02)^b^***	0.41 (0.03)***	0.37 (0.02)***
**Analyzing two scales**						
Mathematics achievement	0.59 (0.02)***	0.61 (0.02)***	0.67 (0.02)***	0.29(0.02)^a^***	0.07 (0.04)	0.40(0.02)***
ERS criterion	0.14(0.03)***	0.03 (0.03)	0.11(0.03)**	0.30 (0.02)^b^***	0.51 (0.02)***	0.47 (0.02)***

a, the point-biserial correlation between mathematics achievement and the latent class factor. b, the point-biserial correlation between the ERS criterion and the latent class factor. The gray cell is the correlation between a factor and its criterion. **p < 0.01; ***p < 0.001.

When these models were fitted to the response to the Mathematics Self-efficacy scale, in all models, a moderate association between mathematics self-efficacy and mathematics achievement (the content criterion) and a clear dissociation between mathematics self-efficacy and the ERS criterion was observed. For comparison, the associations between the criteria and mathematics self-efficacy were investigated by using the PCM, yielding *r* = 0.59 for the relationship between mathematics self-efficacy and its criterion and *r* = 0.30 for the relationship between mathematics self-efficacy and the ERS criterion. The correlation between the content criterion and mathematics self-efficacy in the PCM was virtually identical with that in the mPCM (0.58) and smaller than that in the MNRM (0.64) and the IR tree model (0.67). The correlation between the ERS criterion and mathematics self-efficacy in the PCM (0.30) decreased in the mPCM (0.15) and the IR tree model (0.11) and disappeared in the MNRM (0.01). These results were consistent with Study 1, indicating that the three IRT models could control for the effects of response styles on the mathematics self-efficacy factor.

However, these models did not perform well in measuring ERS. The ERS factors in the three models were related to the ERS criterion (0.27, 0.41, 0.37), but in the mPCM and the IR tree model, the correlation of the ERS factor with the content criterion (0.32, 0.51) was higher than the correlation of the ERS factor with its own criterion (0.27, 0.37). This finding indicates that the ERS factors in the mPCM and the IR tree model might capture more content variance than ERS variance, given that there was not an association between the content criterion and the ERS criterion (0.04).

When the mathematics anxiety scale was added to the analysis, the validity of these models for measuring ERS improved, and they were still valid in controlling the effect of ERS. The relationship of the ERS factor with the content criteria and ERS changed substantially in the MNRM and IR tree model. The ERS factor became more strongly related to its criterion and less weakly related to the content criterion in the MNRM (from 0.41 to 0.51 and from 0.28 to 0.07) and the IR tree model (from 0.37 to 0.47 and from 0.51 to 0.40). The improvement was relatively small in the mPCM. The relation between the ERS factor and its criterion increased from 0.27 to 0.30, and the association between the factor and the content criterion decreased from 0.32 to 0.29.

As the mPCM did not perform well in measuring ERS, its classification quality was checked. When only one scale was analyzed, the entropy and the reduction of classification error were 0.958 and 0.951, respectively. When two scales were analyzed, they were 0.935 and 0.925, respectively. The high value of the classification quality measures suggested that the low performance of the mPCM in measuring ERS might not be due to the classification inaccuracy.

Overall, the three models could disentangle an ERS factor with more ERS variance and less content variance from the responses to two scales than from the responses to one scale.

## General Discussion

There is an increasing interest in response styles (Khorramdel et al., 2019), which can distort responses to rating scales and cause biased results. The mPCM, MNRM, and IR tree model are three widely used approaches for measuring and controlling response styles. The current research examined their validity through two empirical studies. The results consistently showed that these models were effective in correcting the content factor for ERS and MRS effects, and they performed similarly. They could also measure ERS and MRS, though the validity of the mPCM was not good in some cases. Their validity increased when analyzing two scales simultaneously. Overall, the MNRM performed best, followed by the IR tree model.

### Validity of the Three Models for Measuring Extreme and Midpoint Response Styles

The response style factors in the three models were more related to the response style criteria than the content criteria. These results indicate that these models could measure ERS and MRS. However, the correlations were not high, ranging from 0.22 to 0.65. This result was in line with Kieruj and Moors (2013) research. Such a magnitude of correlations indicates the response style factors yielded by these models may not represent participants’ response style levels accurately, given that the response style criteria were obtained through RIRS, which is generally regarded as valid in measuring response styles (e.g., Greenleaf, 1992; De Beuckelaer et al., 2010). Some researchers may argue that this is not true because response styles may be partially domain specific (Cabooter et al., 2017). The response style criteria might be general response style tendencies, while the response style factors might represent domain-specific tendencies. However, in this research, the domain-specific feature of response styles should not be the main cause of the low to medium correlations between response style factors and their criteria because the criteria were computed based on items from the domain same as the target scale.

It is worth noting that the low to medium correlations between the ERS and MRS factors and their criteria do not imply that the models of interest are not valid in measuring ERS and MRS. The short scales in the current research might be insufficient for estimating ERS and MRS because the small number of items might cause high uncertainty (e.g., standard error and classification error) of ERS and MRS estimates in these models (Huang, 2016; Adams et al., 2019). Indeed, the classification performance of the mPCM was low in Study 1, and this might cause the poor efficacy of the mPCM in measuring MRS.

Study 2 suggested that increasing the number of scales might increase the validity of the models of interest in measuring response styles. Firstly, correlations between ERS factors and their criteria increased when these models were fitted to two scales (see Table 9), indicating that the simultaneous analysis of multiple scales might produce ERS factors that contain more ERS variance than analyzing one scale. This finding was consistent with Bolt and Newton (2011) research. Secondly, correlations between ERS factors and the content criterion decreased (see Table 9), indicating that using the three models to fit more than one scale could produce ERS indicators containing less variance of item content.

There were two possible explanations for the improvement in the validity of the models. One is that incorporating more items into the analyses increased the accuracy of ERS estimates. The other is that adding a new scale increased the heterogeneity of items, and the greater heterogeneity made these models less affected by the problem that different combinations of content factors and ERS and MRS could lead to the same response (cf. Böckenholt, 2012; see also Adams et al., 2019). For example, both the combination of a high content factor level plus an intermediate ERS level and the combination of an intermediate content factor level plus a high ERS level can lead to an extreme response. If one participant shows a large proportion of extreme responses, these models have difficulty in distinguishing which combination leads to these responses and yield ERS factors that are confounded with content variance. It was likely that one participant had a large proportion of extreme responses on the Mathematics Self-efficacy scale. However, this was less likely on both the Mathematics Self-efficacy and the Mathematics Anxiety scales because it requires that the mathematics self-efficacy and anxiety factors were at the same level, given that ERS is stable across scales (Wetzel et al., 2013).

The rationales for using the three IRT models to measure ERS and MRS differ greatly. Overall, the results showed that the MNRM performed slightly better than the mPCM and the IR tree model. The reason why the IR tree model performed worse might be that the IR tree model assumes that an extreme response is entirely caused by ERS and a midpoint response is entirely caused by MRS. This causes the IR tree model more susceptible to the problem that different combinations of the level of content factors and the level of response styles could lead to the same response (Böckenholt, 2012; Plieninger and Meiser, 2014).

There were two possible reasons why the MNRM performed better than the mPCM. One was that the MNRM used latent continuous factors to represent ERS and MRS, respectively, while the mPCM used latent categorical factors to represent ERS and MRS. Another possible reason was that the MNRM with the scoring function in the current research was a confirmatory model for detecting response styles, whereas the mPCM was more alike an exploratory model because it did not constrain the order of different threshold parameters within or between subpopulations.

It should be highlighted that the results do not imply that the rationale of the MNRM captures the true response process, but that it may be better to use the MNRM to measure ERS and MRS than the mPCM and the IR tree model. In addition, these models can be modified, and the conclusion may not be generalized to their variants.

### Validity of the Three Models for Controlling Extreme and Midpoint Response Styles

As Studies 1 and 2 showed, the content factor was weakly related to the response style criteria when the assessments were analyzed with the PCM, but such a weak association disappeared when the assessments were analyzed with the models of interest. The results indicated that the three models could yield pure content factor scores without the effects of ERS and MRS.

Study 1 and Study 2 analyzed assessments from two data sets that covered two forms of rating scales (4-point fully labeled scales, 7-point scale with only extreme categories labeled) and two countries (China and Germany), respectively. Previous research revealed that fully labeled scales and the existence of a midpoint led to lower ERS levels (Weijters et al., 2010a), and German had higher ERS levels than Chinese (De Jong et al., 2008). However, the performance of these models for correcting content factor scores for ERS and MRS was always satisfactory across all forms of scales and countries. This finding suggests that the validity of these approaches in controlling ERS and MRS may not be affected by the amount of response style variance in the data. We acknowledge that this needs to be confirmed by more studies.

One influence of response styles on results based on self-report data is that they may distort the magnitude of correlations between variables (e.g., Baumgartner and Steenkamp, 2001; Plieninger, 2016). The reason is that self-report data is prone to the contamination of response styles, and the correlations among scale scores may be partially caused by the effects of response styles. Another impact of response styles is that the differences in response styles among groups may contribute to the differences in variables among these groups (e.g., Moors, 2012; Mottus et al., 2012). Using the three models to analyze self-report data may avoid the above potential influence of ERS and MRS because the current research found that content factor scores yielded by the three IRT models were free of ERS and MRS effects.

### Limitations and Future Directions

This study only investigated ERS and MRS. However, other response styles, such as ARS and DRS, have been found in responses to rating scales (e.g., Weijters et al., 2010b). Future research can examine the validity of the MNRM, the extensions of IR tree models (e.g., Plieninger and Heck, 2018; Park and Wu, 2019), and methods outside the IRT framework (e.g., latent class confirmatory factor models; Moors et al., 2014) for measuring ARS and DRS. Another limitation is that the current research only focused on one definition of ERS and MRS. We acknowledge that adopting different definitions might lead to different results.

Cronbach’s α of the target scale was low (0.67) in Study 1 and high in Study 2 (0.92). Although the results for both studies were similar, Study 2 only investigated ERS. Thus, the low reliability in Study 1 may reduce the generality of the results for the MRS.

The rationales of the three IRT models are different. The results of current research only suggest that which models may perform better in measuring response styles or correcting their effects rather than that the rationale of which model is more reasonable. However, it is critical to understand the nature of response styles and how they and content factors interactively impact the response processes (Khorramdel et al., 2019). Such understanding may help researchers to develop better methods for controlling and measuring response styles.

## Data Availability Statement

The datasets analyzed in Study 1 can be found in the GESIS Data Archive https://dbk.gesis.org/dbksearch/sdesc2.asp?no=5081&db=e&doi=10.4232/1.12509. The datasets analyzed in Study 2 can be found in the PISA official website https://www.oecd.org/pisa/data/pisa2012database-downloadabledata.htm.

## Author Contributions

YZ and YW designed the study. YZ conducted the main analyses and wrote the manuscript. YW provided critical revisions and organization of the manuscript.

## Conflict of Interest

The authors declare that the research was conducted in the absence of any commercial or financial relationships that could be construed as a potential conflict of interest.

## Footnotes

1 The data file version used by Plieninger and Meiser (2014) was 1.0.0. However, the data were updated on April 31st, 2016. We have no access to the old data file. Therefore, this research used the new data file, version 1.1.0.

2 It should be noted that removing pseudo item IV can cause loss of information because it makes categories 1 and 2, as well as 4 and 5, indistinguishable.
