# Supplementary material for: Validity of Three IRT Models for Measuring and Controlling Extreme and Midpoint Response Styles
Source: Front Psychol. 2020 Feb 21;11:271. doi: 10.3389/fpsyg.2020.00271 (PMC7049783; doi:10.3389/fpsyg.2020.00271)
Supplement: Supplementary file 1 [file Data_Sheet_1.docx]

# Appendix

Vermunt (2010) suggested using the reduction of classification error to characterize the classification performance of a latent class model. To calculate this measure, we first need to calculate the estimated average classification error given by

$$E= \sum_{j=1}^{N} \frac{1}{N}\left\{ 1-max\left[ P\left( v_{j}=c|\mathbf{Y}_{\mathbf{j}} \right) \right] \right\} \left( A1 \right).$$

*N* is the number of cases. $P\left( v_{j}=c|\mathbf{Y}_{\mathbf{j}} \right)$ is the posterior probability that individual *j* belongs to the latent class *c*, given the observed response patterns $\mathbf{Y}_{\mathbf{j}}$. By comparing *E* and the classification error based on the unconditional probabilities $P\left( v_{j}=c \right)$, which is exactly $\pi_{c}$ in equation 1, we get the reduction of classification error given by

$$\lambda=1-\frac{E}{1-max\left[ P\left( v_{j}=c \right) \right]} \left( A2 \right).$$

The closer $\lambda$ is to 1, the better the classification quality of a model.

**References**

Vermunt, J. K. (2010). “Latent class models,” in *International Encyclopedia of Education*, Vol. 7, eds P. Peterson, E. Baker, and B. McGaw, (Oxford: Elsevier), 238–244.
